# Item-specific control of word reading as evidenced by a novel proportion wordness effect

**DOI:** 10.3758/s13421-026-01876-4

**Published:** 2026-04-28

**Authors:** Eldad Keha, Julie M. Bugg

**Affiliations:** 1 Department of Psychology, The Hebrew University of Jerusalem, Mt. Scopus, 91905 Jerusalem, Israel; 2 Department of Psychology, Achva Academic College, Beer Tuvia, Israel; 3 Department of Psychological & Brain Sciences, Washington University in St. Louis, St. Louis, MO USA

**Keywords:** Item-specific proportion congruence, Item-specific proportion wordness, Cognitive control, Stroop task, Task control

## Abstract

Jacoby and colleagues demonstrated that control processes can adapt at the level of individual stimuli, giving rise to the item-specific proportion congruence (ISPC) effect, whereby items that frequently appear as incongruent produce a smaller Stroop effect. Prior research has focused exclusively on *informational conflict* (competition between word meaning and ink color), leaving open whether item-specific control mechanisms also affect *task conflict* (competition between the task sets of word reading and color naming). Across two experiments, we addressed this question. In Experiment 1, using an ISPC manipulation, we replicated the classic reduction of informational conflict for mostly incongruent items but found no evidence of item-specific control of task conflict. In Experiment 2, we introduced a novel item-specific proportion wordness (ISPW) manipulation to vary the likelihood of task conflict at the item level, by presenting items mostly with neutral words or neutral nonwords. Results revealed robust item-specific effects on task conflict and informational conflict, with reductions in both types of conflict for items that were mostly neutral words. These findings clarify the scope of item-specific control: while manipulations of reactive informational control selectively influence informational conflict, manipulations of reactive task control can regulate not only task conflict but additionally informational conflict. Our results refine and extend Jacoby et al.’s original insight, providing initial evidence for item-specific control of word reading, and demonstrating the operation of item-specific control at multiple levels of conflict via an ISPW manipulation that modulated the likelihood of word reading.

**Supplementary Information:**

The online version contains supplementary material available at https://doi.org/10.3758/s13421-026-01876-4.

In a seminal contribution, Jacoby and colleagues (2003) challenged extant conceptualizations of control by demonstrating that control may adapt not only to the general conditions of a task but also to statistical regularities tied to specific items. They showed that when items (in their case, certain words in a color–word Stroop task) were most often paired with an incongruent (as compared with congruent) color, the Stroop effect was reduced as if an automatic form of control had minimized the distracting potential of the word for mostly incongruent items. This item-specific proportion congruence (ISPC) effect was striking because it suggested that control is not always a slow and deliberate mechanism that applies generally to all stimuli; instead, it can operate rapidly and flexibly, reactively adjusting post-stimulus onset to the frequency with which specific items tend to elicit conflict. The discovery of the ISPC effect has stimulated much subsequent research that confirmed Jacoby’s suggestion that item-specific control reflects contributions of control and memory processes (i.e., control; Bugg, 2015; Dey et al., 2025; Ileri-Tayar, Bugg et al., 2025a; Spinelli & Lupker, 2020; associative, contingency learning; see Schmidt & Besner, 2008; episodic retrieval; Bugg & Crump, 2012; Crump & Milliken, 2009; Gallego et al., 2025) and may indeed operate automatically (Suh & Bugg, 2021; Ileri-Tayar, Bugg, et al., 2025a; Ileri-Tayar et al., 2022, 2024). The present study harnesses foundational research on the ISPC effect along with a set of theoretical advances that happened in parallel—namely, evidence showing that multiple types of conflict contribute to the Stroop effect (e.g., informational and task conflict; Augustinova et al., 2019; Goldfarb & Henik, 2007; Keha et al., 2025; Keha & Kalanthroff, 2025a, b; Littman et al., 2019; Shichel & Tzelgov, 2018) to address the following question: Does item-specific control reflect a general mechanism that operates on all forms of conflict, or does it selectively influence certain types of conflict? Before detailing the two types of conflict of interest in the present study, we first provide some additional background on the ISPC effect, including underlying mechanisms.

One of the most widely used tasks to examine the ISPC effect is the color–word Stroop task (Atalay & Misirlisoy, 2012; Bugg & Hutchison, 2013; Ileri-Tayar, Bugg, et al., 2025a; Ileri-Tayar et al., 2022, 2024; Jacoby et al., 2003; Spinelli & Lupker, 2020), where participants name the ink color of a color word. A congruency (i.e., Stroop) effect reliably emerges, indicating slower (and sometimes more errant) responding on incongruent trials where the color conflicts with the word (e.g., RED in blue ink) than on congruent trials where the color matches the word (e.g., BLUE in blue ink). In recent years, many studies have adopted a confound-minimized ISPC design where colors (rather than words) predict conflict frequency (thereby holding constant the contingency between the PC-predicting dimension [color] and responses) and all colors appear with all words such that there are three equally likely responses on incongruent trials (Bugg & Dey, 2018; Bugg & Hutchison, 2013; Bugg et al., 2011; Chiu & Egner, 2017; Ileri-Tayar, Bugg et al., 2025a; Ileri-Tayar et al., 2022, 2024; see Suh & Bugg, 2021, for discussion of potential confounds; see Braem et al., 2019, for a consensus paper). For example, the colors red and yellow might be mostly congruent, appearing in a congruent format on most trials (e.g., RED in red ink) and only occasionally in an incongruent format (i.e., YELLOW, BLUE, or GREEN in red ink). In contrast, the colors blue and green might be mostly incongruent, appearing in an incongruent format on most trials and only occasionally in a congruent format. As in the original design, an ISPC effect is observed showing that the congruency effect is reduced for mostly incongruent items compared with mostly congruent items.

One prominent explanation for the ISPC effect is based on the episodic retrieval account (Crump & Milliken, 2009; for extension to the ISPC, see Bugg & Crump, 2012). On this view, each encounter with an item (stimulus color) is encoded in memory together with the control setting that was engaged on that trial. A control setting might be relatively focused, assigning less weight to the word dimension (and/or more to the color dimension), as is anticipated for incongruent trials, or relatively relaxed, with some weight given to the word dimension, for congruent trials. When the item is presented again, it cues retrieval of these prior episodes, reinstating the associated control state. This retrieval process dynamically biases how attention is allocated; for mostly incongruent items (colors that have frequently appeared in incongruent trials with a focused control state), retrieval reinstates stronger suppression of the word dimension than mostly congruent items (colors that have frequently appeared in congruent trials with a relaxed control state). Thus, the ISPC effect emerges because each stimulus reactivates a memory trace of prior episodes, effectively carrying forward the item’s history of conflict and control setting into performance on the current trial (for alternative accounts see Schmidt & Besner, 2008; Gallego et al., 2025). The ISPC effect provides a striking example of reactive control (engaged after conflict is detected) rather than proactive control (engaged in advance to prevent conflict; Braver, 2012; Gonthier et al., 2016; Spinelli et al., 2022). For this reason, researchers have employed the ISPC manipulation to examine a range of questions, such as whether reactive control is impaired (or not) in aging (Ball et al., 2023; Bugg, 2014; Ileri-Tayar, Suh et al., 2025c) and what the neural mechanisms are that uniquely give rise to reactive control (Braver et al., 2021; Grandjean et al., 2013; Shedden et al., 2013).

All prior research utilizing the ISPC manipulation has examined how an item’s conflict history influences informational conflict. Informational conflict arises when the task-relevant ink color and the task-irrelevant word compete, as in incongruent trials. Informational conflict is typically measured by the *congruency effect* (i.e., Stroop effect), the slowing that is observed when comparing incongruent to congruent stimuli (see Table 1). Conceptually speaking, informational conflict arises from two possible sources: response competition between the responses that are activated by the color and word dimensions and semantic competition between their meanings (Shichel & Tzelgov, 2018). However, there is also evidence for a different type of conflict in the Stroop task called task conflict, reflecting competition between the task sets of word reading and color naming. Importantly, task conflict, unlike informational conflict, can emerge on congruent or neutral word trials, in addition to incongruent trials, because all of these stimulus types comprise stimuli that are readable (Aarts et al., 2009; Hershman et al., 2021). Task conflict is measured by the *word interference effect*, the slowing that is observed when comparing word (readable) stimuli to nonword (non-readable) stimuli (Keha et al., 2025; Keha & Kalanthroff, 2025a, b; Kinoshita et al., 2018; Mills, 2017; see Table 1). While it is now well established that Stroop performance indexes not only response or semantic competition between stimulus dimensions (informational conflict) but also a broader struggle between relevant and irrelevant task sets (task conflict), it is not yet known whether item-specific control mechanisms operate on task conflict or solely informational conflict.

**Table 1:** Operational definitions of informational and task conflict markers in the Stroop task

Name of marker	Contrast	Example	Conflict
Congruency effect	Incongruent–Congruent	RED_yellow_ –RED_red_	Informational
Word interference effect	Neutral words–Neutral nonwords	TREND_red_–####_red_	Task

*Note.* The table summarizes the two conflict markers that were used in the current study, including the name of each marker, the contrast used to compute it, and an illustrative example. In the examples, the word is capitalized, and the ink color is indicated using a subscript.

## Present Study

The present study was comprised of two experiments. The major aim of Experiment 1 was to investigate for the first time whether the ISPC manipulation affects informational conflict (measured by the congruency effect) exclusively or additionally affects task conflict (measured by the word interference effect). Turning back to Jacoby and colleagues (2003), one of the conclusions from their seminal ISPC study was that “results suggest automatic control of automatic processes (word reading)” (p. 643). Although some studies have provided evidence for the automaticity of item-specific control through manipulations of load and transfer (Ileri-Tayar et al., 2022, 2024; Ileri-Tayar, Suh et al., 2025c; Suh & Bugg, 2021), it remains unknown whether what is being automatically controlled is informational conflict or task conflict. No study has examined whether word reading is amenable to item-specific control because no study has measured task conflict. One might posit that the typical ISPC pattern in the confound-minimized design, a large difference in performance on incongruent trials (i.e., faster responses for incongruent trials for mostly incongruent items than mostly congruent items) with little and often no effect on congruent trials, casts doubt on this possibility. In other words, if this typical pattern occurs because frequently encountering conflict at the item level leads to a reduction in word reading, resulting in faster responding on incongruent trials for mostly incongruent compared with mostly congruent items, then it stands to reason that a similar speeding should be observed on congruent trials, which also afford reading. However, this is speculative and not a strong test because prior studies lacked the appropriate control condition to assess task conflict (a nonreadable stimulus). Addressing this question is theoretically important because it speaks to the scope of item-specific control, and more generally, properties of reactive control. If the ISPC manipulation influences conflict resolution across multiple levels of processing (informational conflict and task conflict), this pattern would suggest the scope of item-specific control is broader than previously documented, supporting Jacoby’s speculation about control over word reading.

## Experiment 1

Many prior studies have demonstrated that the ISPC manipulation reliably affects the magnitude of the congruency effect, a marker of informational conflict. Experiment 1 examined whether this manipulation also extends to task conflict—that is, to the control of word reading. Using the confound-minimized ISPC manipulation linked to specific colors (mostly congruent vs. mostly incongruent colors), we asked whether the reduced congruency effect routinely observed for mostly incongruent items would be accompanied by a reduction in the word interference effect, reflecting a modulation of task conflict. To measure task conflict, we incorporated neutral words and neutral nonwords in the ISPC paradigm and held the frequencies of these two types of trials constant between the mostly congruent and mostly incongruent colors. This approach allowed us to measure whether a manipulation of reactive informational control affects task conflict as measured by the word interference effect.^1^ A reduction in the word interference effect for mostly incongruent compared with mostly congruent items would imply that the same item-specific adjustments that reduce informational conflict (response and/or semantic competition) also reduce task conflict (the competition between the irrelevant but more automatic task of word reading and the task of color naming).

### Method

#### Participants

The experiment was preregistered at the following link: https://osf.io/gjd8s. The sample size was determined based on Bugg and Hutchison (2013, Experiment 1), who reported a partial Eta squared effect size of 0.67 for the ISPC effect in a color–word Stroop task using the confound-minimized design. Given that effect sizes for task conflict are generally smaller (about 2–3 times smaller; see Keha & Kalanthroff, 2025a; Kinoshita et al., 2018), we decided to triple the sample size used by Bugg and Hutchison, resulting in a target of 60 participants instead of 20. We collected data from 66 Achva Academic College students who participated in the study to fulfill a course requirement in psychology. All participants were native Hebrew speakers with normal or corrected-to-normal vision and normal color vision. Five participants were excluded due to technical problems (the microphone failed to detect responses), two due to low accuracy (more than 3 standard deviations below the group's mean accuracy), and one due to slow reaction times (more than 3 standard deviations above the group's mean reaction time). Thus, the analyzed sample included 58 participants (51 women and seven men; mean age = 22.88 years, *SD* = 2.06). To address the sensitivity of the analyzed sample size, we conducted a sensitivity power analysis using MorePower (Campbell & Thompson, 2012) for a 4 × 2 repeated-measures analysis of variance (ANOVA), with α = .05. Results indicated that a sample size of *N* = 58 achieves 80% power to detect a partial eta squared effect of ${\upeta }_{p}^{2}$= .061 (equivalent to Cohen’s *f* = .255).

#### Design and stimuli

We employed a 4 (stimulus type: congruent, incongruent, neutral words, neutral nonwords) × 2 (item type: mostly congruent vs. mostly incongruent) within-subjects design. The stimuli consisted of Hebrew words displayed in Arial 36 font, each presented in one of four colors (red, yellow, blue, green). The word stimuli included four color words (RED, YELLOW, BLUE, GREEN), two neutral words (TREND, BUILDING), and two neutral nonwords (~~~~,####), and all were four characters long in Hebrew. Congruent trials involved words appearing in their corresponding color (e.g., RED in red ink), and incongruent trials involved words appearing in a different color (e.g., RED in green ink). The frequencies of each stimulus-color combination within a single experimental block are summarized in Table 2. Two colors were assigned to the mostly congruent item type. For these colors, trials were congruent on 55% of occurrences, and the remaining trials were evenly distributed across the other three stimulus types, such that each color appeared as incongruent on 15%, as a neutral word on 15%, and as a neutral nonword on 15% of trials. The other two colors were assigned to the mostly incongruent item type. For these colors, trials were incongruent on 55% of occurrences, and each color appeared as congruent on 15%, as a neutral word on 15%, and as a neutral nonword on 15% of trials. The assignment of specific colors to item types (mostly congruent vs. mostly incongruent) was counterbalanced across participants.

**Table 2:** Frequency of color–word pairings for mostly congruent and mostly incongruent items in Experiment 1

	Color			
Word	Red	Yellow	Blue	Green
RED	54	2	18	18
YELLOW	2	54	18	18
BLUE	2	2	6	18
GREEN	2	2	18	6
TREND	3	3	3	3
BUILDING	3	3	3	3
~~~~	3	3	3	3
####	3	3	3	3

Note. The frequencies reflect the composition of a single block. In this example, the colors red and yellow are mostly congruent items, and the colors blue and green are mostly incongruent items

#### Procedure

The study was approved by the Institutional Review Board at Achva Academic College. We used E-Prime 3.0.3.184 software (PST; Psychological Software Tools, Pittsburgh, PA) to present stimuli and collect responses, which were recorded vocally using a microphone connected to an E-Prime Chronos response box. Participants sat approximately 60 cm from the monitor, with the experimenter seated next to them to record their vocal response via keyboard. Participants were instructed to name aloud the ink color, not the word itself, and to respond as quickly and accurately as possible. The task consisted of 864 trials, equally divided into three blocks of 288 trials each. Each block contained 120 congruent trials, 120 incongruent trials, 24 neutral word trials, and 24 neutral nonwords trials, presented in random order. Each trial began with a 500-ms black fixation cross followed by the stimulus (both were centrally presented on a grey background), which appeared for 2,000 ms or until a vocal response was detected. E-Prime recorded the RT associated with the vocal response. After the participant responded, the experimenter used a keyboard to code what color they spoke aloud first so that accuracy could be derived (e.g., if the participant said “blue,” the experimenter pressed the key marked blue). The next trial started 2,000 ms after the participant's vocal response. Scratch trials were trials in which the voice-key was tripped by extraneous noise or imperceptible speech. The experimental trials were preceded by 20 practice trials mirroring the proportions of trials in the experimental block. Participants were thanked and debriefed on the study before departing.

### Results

**Fig. 1:**
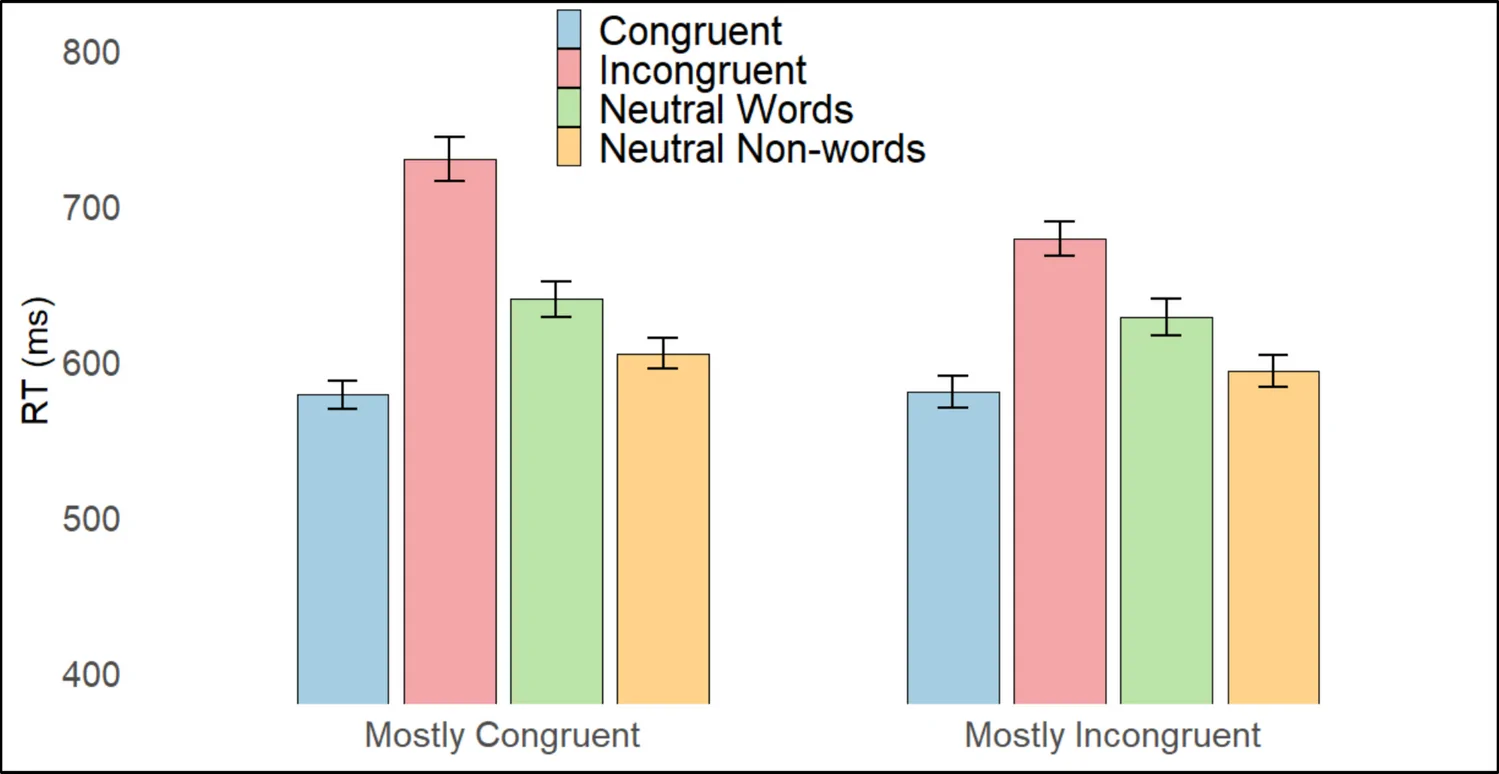
Mean reaction times for all stimulus types in the mostly congruent and mostly incongruent conditions for Experiment 1. Reaction times (RTs) for congruent, incongruent, neutral word, and neutral nonword trials as a function of item type. Bars depict mean RTs (in ms) for mostly congruent items (left panel) and mostly incongruent items (right panel). Error bars represent 1 standard error below/above the mean. (Color figure online)

We carried out a 4 × 2 ANOVA for reaction time (RT), with stimulus type (congruent, incongruent, neutral words, neutral nonwords) and item type (mostly congruent, mostly incongruent) as within-subject factors (see Fig. 1 and Table S3 in the Supplementary Materials). Incorrect trials (around 10.05% of all trials) and scratch trials (0.57% of all trials) were excluded from the analysis. In addition, trials that were faster than 200 ms were removed from the analysis (e.g., Bugg & Hutchison, 2013; Bugg et al., 2011; Ileri-Tayar et al., 2022; Suh & Bugg, 2021). This exclusion resulted in the removal of 617 trials, which is approximately 1.37% of the total number of correct trials. The exact same 4 × 2 ANOVA was carried out on error rates. Error rate was low (*M* = 7.3, *SD* = 4.8) and the pattern of the analysis was in line with the reaction time analysis (see for further details the Supplementary Materials).

For the RT analysis, we observed a main effect of stimulus type, *F*(3,171) = 205.079, *p* < .001, ${\upeta }_{p}^{2}$ = .783. To determine whether the markers of informational conflict and task conflict were evident, we conducted pairwise contrasts using a Benjamini–Hochberg correction. There was evidence for the informational conflict marker, the congruency effect, with slower RTs for incongruent than congruent trials, *t*(57) = 18.807, *p* < .001, Cohen’s *d* = 2.491. There was also evidence for the task conflict marker, the word interference effect, with slower RTs for neutral words compared with neutral nonwords, *t*(57) = 9.697, *p* < .001, Cohen’s *d* = 1.285. In addition, we observed a main effect of item type, *F*(1,57) = 26.972, *p* < .001, ${\upeta }_{p}^{2}$= .321, with RTs being significantly faster for mostly incongruent items compared with mostly congruent items.

More importantly, we observed a two-way interaction between stimulus type and item type *F*(3,171) = 22.040, *p* < .001, ${\upeta }_{p}^{2}$ = .279. We decomposed this interaction with targeted contrasts using Benjamini–Hochberg corrections to address two questions. The first question was whether the ISPC manipulation affects informational conflict. The congruency effect (incongruent vs. congruent) was significantly smaller for mostly incongruent items (*M* = 98, *SE* = 6) than for mostly congruent items (*M* = 151, *SE* = 9), *t*(57) = 7.145, *p* < .001, Cohen’s *d* = 0.946, replicating many prior studies. The second question was whether the ISPC manipulation affects task conflict. There was no effect of the ISPC manipulation on the word interference effect (neutral words vs. neutral nonwords), which did not differ between mostly congruent (*M* = 35, *SE* = 5) and mostly incongruent (*M* = 35, *SE* = 5) items, *t*(57) = 0.056, *p* = .956).^2^

### Discussion

Experiment 1 demonstrated the typical effect of the ISPC manipulation on informational conflict, with a smaller congruency effect observed for mostly incongruent than mostly congruent items. However, the ISPC manipulation did not affect task conflict (as there were no changes in the word interference effect), and this finding runs counter to the suggestion of Jacoby et al. (2003) that the ISPC effect can be interpreted as indicating control over word reading. One potential explanation for this pattern is that task conflict is simply not amenable to reactive modulation at the item level. An alternative explanation is that task conflict is amenable to reactive modulation at the item level, but modulations in task control over word reading are not triggered by an item’s history of informational conflict (i.e., how often stimuli are incongruent versus congruent). Accordingly, it may be that item-level manipulations of a given conflict type (e.g., informational conflict) selectively influence control over that conflict type (e.g., informational conflict).

## Experiment 2

In Experiment 1, the ISPC manipulation did not provide item-level cues about the costs of engaging the reading task itself because readable words occurred equally often for mostly congruent and mostly incongruent items. In Experiment 2 we tested the novel prediction that item-specific control over task conflict should emerge when the item-specific manipulation targets *word reading* rather than congruency. To do so, we introduced a novel item-specific proportion “wordness” (ISPW) manipulation. For one subset of colors, trials were mostly neutral words, whereas for another subset of colors, trials were mostly neutral nonwords, while holding constant the proportion of congruent and incongruent trials for these colors. This manipulation creates item-level cues about whether engaging the reading task is likely to be costly. If item-specific control can leverage these cues to regulate task conflict, then colors frequently paired with readable words should elicit stronger task-control settings and, consequently, a smaller word interference effect. Moreover, to the extent that reduced engagement with word reading limits distractor processing, stronger task control for mostly word colors may secondarily attenuate informational conflict.

### Method

#### Participants

The same power analysis used in Experiment 1 (based on Bugg & Hutchison, 2013, Experiment 1; target *N* = 60) guided sampling for Experiment 2. Anticipating higher exclusion rates online, we recruited 81 participants via Prolific and compensated them approximately £2 for participation. Participants provided informed consent prior to participation. All participants self-reported being English speakers with normal or corrected-to-normal vision and normal color vision, and were between 18 and 40 years of age. Data from 10 participants were excluded due to a failure to complete the task, and three participants due to technical problems. Another three participants were excluded for having low accuracy (more than 3 standard deviations below the group mean). Therefore, the analyzed sample comprised 65 participants (31 women, 34 men; *M*_age_ = 32.29 years, *SD* = 5.62).

#### Design and stimuli

We employed a 4 (stimulus type: congruent, incongruent, neutral words, neutral nonwords) × 2 (item type: mostly neutral words vs. mostly neutral nonwords) within-subjects design. Stimuli were English words presented in one of four ink colors (blue, green, red, yellow). The color words were BLUE, GREEN, RED, and YELLOW. Neutral words were MASK, TREND, LOCKER, and PUT. Neutral nonwords were ~~~~, ####, ****, and %%%%. All stimuli were uppercase in Arial 36-point font, and each appeared in all four colors. The item-specific proportion manipulation targeted task control via “wordness”: Two colors were assigned to the mostly neutral words item type. For these colors, trials were neutral words on 50% of occurrences, and the remaining trials were evenly distributed across the other three stimulus types, such that each color appeared as a neutral nonword on 16.67%, congruent on 16.67%, and incongruent on 16.67% of occurrences. The other two colors were assigned to the mostly neutral nonwords item type. For these colors, trials were neutral nonwords on 50% of occurrences, and each color appeared as a neutral word on 16.67%, congruent on 16.67%, and incongruent on 16.67% of occurrences. The assignment of specific colors to item types (mostly neutral words vs. mostly neutral nonwords) was counterbalanced across participants.

#### Procedure

The procedure was the same as in Experiment 1, with two exceptions:

1. A manual task instead of a vocal task was used (including a 20 trials color-mapping practice block), with the keys C, V, B, and N corresponding to red, green, blue, and yellow colors, respectively;
2. There were two blocks of 216 trials each (432 trials in total; see Table 3 for frequency of color–word pairings), and in each block, there were 72 neutral word trials, 72 neutral nonword trials, 36 congruent trials, and 36 incongruent trials.

**Table 3:** Frequency of color–word pairings for mostly neutral words and mostly neutral nonwords items used in Experiment 2

	Color			
Word	Blue	Green	Red	Yellow
BLUE	9		9	
GREEN	9	9		
RED			9	9
YELLOW		9		9
~~~~			27	
####	9			
****		9		
%%%%				27
MASK			9	
TREND	27			
LOCKER		27		
PUT				9

Note. The frequencies reflect the composition of a single block. In this example, the colors blue and green are mostly neutral words items, and the colors red and yellow are mostly neutral nonwords items

### Results

**Fig. 2:**
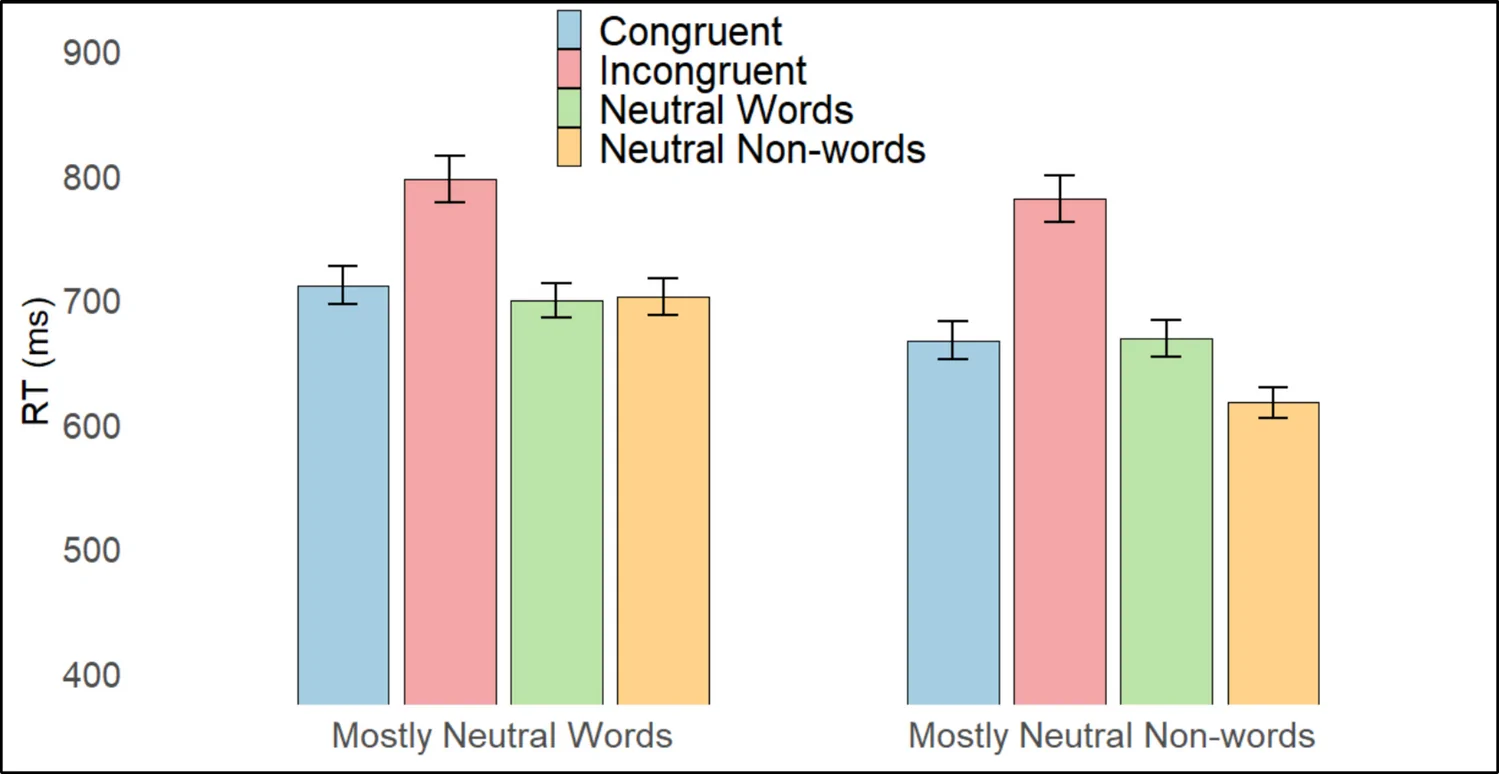
Mean reaction times for all stimulus types in the mostly neutral words and mostly neutral nonwords conditions for Experiment 2. Reaction times (RTs) for congruent, incongruent, neutral word, and neutral nonword trials as a function of item type. Bars depict mean RTs (in ms) for mostly neutral words items (**left panel**) and mostly neutral nonwords items (**right panel**). Error bars represent 1 standard error below/above the mean. (Color figure online)

We carried out a 4 × 2 ANOVA for reaction time with stimulus type (congruent, incongruent, neutral words, neutral nonwords) and item type (mostly neutral words, mostly neutral nonwords) as within-subject factors (see Fig. 2 and Supplementary materials, Table S3). Incorrect trials (around 7.03% of all trials) were excluded from the analysis. In addition, trials that were faster than 200 ms were removed from the analysis. This exclusion resulted in the removal of four trials, which is approximately 0.01% of the total number of correct trials. The exact same 4 × 2 ANOVA was carried out on error rates. Error rate was low (*M* = 6.8, *SD* = 2.8) and the pattern of the results was in line with the reaction time analysis (for further details see the Supplementary Materials).

For the RT analysis, we observed a main effect of stimulus type, *F*(3,192) = 109.410, *p* < .001, ${\upeta }_{p}^{2}=.631$, and conducted post-hoc pairwise contrasts using a Benjamini–Hochberg correction. We begin with the task conflict marker because the ISPW manipulation used in Experiment 2 targeted task conflict. The word interference effect was significant, such that neutral words were slower as compared with neutral nonwords, *t*(64) = 5.202, *p* < .001, Cohen’s *d* = 0.650. The informational conflict marker, the congruency effect, was also significant, showing a slower reaction time in incongruent than congruent trials, *t*(64) = 11.445, *p* < .001, Cohen’s *d* = 1.430. In addition, we observed a main effect of item type, *F*(1,64) = 48.320, *p* < .001, ${\upeta }_{p}^{2}=.430$, with RTs being significantly faster for items that were mostly neutral nonwords compared with items that were mostly neutral words.^3^

More importantly, the main effect of stimulus type that showed an overall significant congruency effect and word interference effect, was qualified by a two-way interaction between stimulus type and item type, *F*(3,192) = 17.418, *p* < .001, ${\upeta }_{p}^{2}=.214$. To decompose this interaction, we examined whether the different markers of conflict differed between the two item type conditions (mostly neutral words vs. mostly neutral nonwords). All the post hoc contrasts included a Benjamini–Hochberg correction. The task conflict marker, the word interference effect (neutral words vs. neutral nonwords), was significantly smaller for the mostly neutral words condition (*M* = −3, *SE* = 6) than the mostly neutral nonwords condition (*M* = 52, *SE* = 6), *t*(64) = 6.143, *p* < .001, Cohen’s *d* = 0.768.^4^ Similarly, the informational conflict marker, the congruency effect (incongruent vs. congruent), was significantly smaller in the mostly neutral words condition (*M* = 85, *SE* = 10) compared with the mostly neutral nonwords condition (*M* = 114, *SE* = 10), *t*(64) = 2.544, *p* = .020, Cohen’s *d* = 0.318.

### Discussion

Experiment 2 tested whether task conflict is modulated by item-specific control by varying the likelihood that a given color is paired with a readable neutral word versus a nonreadable neutral nonword. The results provided clear evidence of item-specific modulation of task conflict. The word-interference effect (neutral words vs. neutral nonwords) was smaller for mostly neutral word colors than for mostly neutral nonword colors, indicating reduced task conflict for colors that frequently co-occur with words. This pattern is consistent with the idea that these colors come to trigger a stronger task-control setting that limits engagement with the irrelevant reading task.

Importantly, the ISPW manipulation also affected informational conflict. The congruency effect (incongruent vs. congruent) was smaller for mostly neutral words than for mostly neutral nonwords, even though the proportions of congruent and incongruent trials were held constant across item types. This suggests that item-specific adjustments linked to task conflict can carry downstream to reduce informational conflict as well.

## General discussion

In their seminal work, Jacoby and colleagues (2003) demonstrated that control processes can adapt at the level of individual items, laying the foundation for decades of research on ISPC effects. Building on this, the present study asked whether such item-specific control adjustments, traditionally demonstrated for informational conflict, also extend to task conflict. In Experiment 1, we replicated prior findings showing that the ISPC manipulation reliably reduced informational conflict, as evidenced by smaller congruency effects for mostly incongruent items. However, the novel finding was that the manipulation did not modulate task conflict, counter to the interpretation of Jacoby et al. (2003) of a mechanism exerting automatic control over word reading.

In Experiment 2, we employed a novel ISPW manipulation by varying the probability of encountering a readable stimulus at the item level and found the first evidence for an item-specific “wordness” effect, showing that task conflict can be modulated at the item level. This novel finding demonstrates that item-specific adjustments extend to the task level, influencing the extent to which words trigger the irrelevant task of reading. Critically, this provides the first demonstration that control over the competition between the task sets of color naming and word reading can be engaged reactively in response to item-level cues. In this way, our work extends Jacoby et al.’s (2003) demonstration of item-level adaptation, highlighting that the scope of such adaptive control may be broader than originally conceived. Specifically, Jacoby’s insight that control can operate item by item is supported, but our data suggest that this control does not automatically suppress word reading based on conflict history (ISPC). Rather, it modulates reading only when items cue the likelihood of engaging the irrelevant task (i.e., task conflict), as in the ISPW manipulation.

In both experiments, we found evidence that informational conflict, as measured by the congruency effect, was modulated by item-specific control, whether the manipulation targeted informational control (ISPC; Experiment 1) or task control (ISPW; Experiment 2). The results of Experiment 1 replicated previous demonstrations that informational conflict is reduced when informational control is high at the item level (i.e., for mostly incongruent items; e.g., Atalay & Misirlisoy, 2012; Bugg & Dey, 2018; Bugg & Hutchison, 2013; Bugg et al., 2011; Ileri-Tayar, Bugg et al., 2025a, Ileri-Tayar, Colvett et al., 2025b; Spinelli & Lupker, 2020). The results of Experiment 2 are novel in demonstrating that informational conflict is also reduced when task control is high at the item level (i.e., for mostly neutral word items). That is, manipulating the likelihood that a color will be associated with a readable word, thereby influencing control over the task of word reading, led to differences in the congruency effect, suggesting transfer of task control to informational conflict.

The finding that ISPW, as manipulated via the proportion of neutral words/nonwords, reduced the congruency effect (akin to an ISPC effect) is also notable because the proportion of congruent and incongruent trials was equated between the mostly neutral words and the mostly neutral nonwords conditions in Experiment 2. This suggests that modulations of task control at the item level affect informational control under conditions that cannot be explained by alternative accounts of ISPC effects, including associative learning (e.g., contingency learning; Schmidt & Besner, 2008) and episodic distractor-driven retrieval accounts (Gallego et al., 2025). Instead, the findings lend further support to the view that item-specific modulations of the congruency effect reflect control mechanisms, such as those emphasized in the episodic retrieval account (e.g., Bugg & Crump, 2012; Crump & Milliken, 2009; see also Bugg & Hutchison, 2013; Ileri-Tayar et al., 2022, 2024; Suh & Bugg, 2021, for further evidence).

Considered together, the findings from Experiments 1 and 2 suggest that the expression of item-specific adjustments depends on which item-level regularity is manipulated. When the item-level regularity concerned congruency (ISPC), the effect was evident on the informational-conflict marker (reduction in the congruency effect for mostly incongruent items) but did not extend to the task-conflict marker (no difference in word interference effect between mostly incongruent and mostly congruent items). When the item-level regularity concerned “wordness” (ISPW), the effect was evident on the task-conflict marker (reduction in the word interference effect for mostly neutral word items) and it extended to the informational-conflict marker (reduction in the congruency effect for the same item type).

### Broader implications

Our findings also have important implications for the proactive control/task conflict (PC-TC) model proposed by Kalanthroff and colleagues (2018). According to this model, task conflict emerges primarily under conditions of weak proactive control, whereas when proactive control is strong, task conflict does not arise or is resolved quickly, minimizing the need for reactive control. In contrast, the present results suggest that task conflict can occur even under conditions of moderate proactive task control, and control over task conflict can operate not only proactively but also reactively. In our ISPW paradigm, words of any type (congruent, incongruent, and neutral words) comprised approximately 67% of all trials, creating a context in which proactive task control should be relatively high. Yet, despite this, we observed item-specific reactive control of task conflict in Experiment 2, demonstrating that reactive engagement is not limited to situations of weak proactive control but can also operate when proactive resources are moderately engaged. This observation calls for an extension of the PC–TC model to accommodate the possibility that reactive control can supplement proactive regulation rather than being confined to low control states. Prior research demonstrated that proactive manipulations of task control, such as list-wide proportion wordness, modulate task conflict (e.g., Goldfarb & Henik, 2007; Keha & Kalanthroff, 2023, 2025a; Parris, 2014; Tzelgov et al., 1992). Our findings extend this body of work by providing the first evidence that task conflict, like informational conflict, can also be regulated through reactive control processes when the likelihood of engaging the irrelevant task is signaled at the item level.

A second implication of our results concerns the relationship between task-level manipulations of control and their downstream effects on informational conflict. Previous research has shown that proactive manipulations of informational control, such as varying the proportion of incongruent trials, can influence both informational conflict and task conflict (Entel et al., 2015). In contrast, our results indicate that reactive manipulations of informational control at the item level did not modulate task conflict, but instead only influenced informational conflict. Thus, while proactive adjustments in informational control can cascade to both types of conflict, reactive adjustments appear to be more specific, targeting informational conflict without reducing the tendency of words to trigger the competing task set. Moreover, the present findings suggest an asymmetric pattern within reactive control processes. When reactive control was engaged at the informational level (Experiment 1), only informational conflict was modulated, whereas engaging reactive control at the task level (Experiment 2) was accompanied by modulation of both task and informational conflict. This asymmetric pattern suggests that within reactive control dynamics, task control operates at a broader and earlier stage of processing that can cascade to influence informational conflict, whereas informational control adjustments occur later (possibly triggered by response conflict) and do not propagate upward to affect task conflict. Consistent with the PC–TC model (Kalanthroff et al., 2018), task conflict represents the initial competition between task sets, such as color naming and word reading, which precedes the emergence of informational conflict. Accordingly, reactive adjustments at the task level can constrain or prevent informational conflict, but reactive adjustments at the informational level cannot retroactively alter task-level activation. A question that merits further investigation is whether this is unique to reactive adjustments. Some studies have shown that information conflict is reduced in conditions in which proactive task control is high (Entel et al., 2015; Kinoshita et al., 2018), but others found no such effect (Goldfarb & Henik, 2007; Keha & Kalanthroff, 2023).

Task conflict, as defined in the present study, has a clear analogue in the task-switching literature on mixing costs. Mixing costs are typically defined as the performance decrement on task-repetition trials in mixed-task blocks relative to repetition trials in single-task blocks, and are commonly attributed to the need to maintain and regulate multiple competing task sets (Shahar & Meiran, 2015). Importantly, task-switching research shows that these costs are not merely global working-memory costs, but are strongly tied to stimulus-triggered task conflict, particularly when stimuli afford more than one task (i.e., bivalent stimuli; Moretti et al., 2024, 2025). This stimulus-based account maps directly onto the Stroop task: readable stimuli afford both the color-naming task and the competing word-reading task, thereby eliciting task conflict in a manner analogous to bivalent stimuli in task switching. From this perspective, the ISPW manipulation used in Experiment 2 might be viewed as an item-specific manipulation of stimulus valency, whereby certain colors more reliably cue stimuli that activate competing task sets. This framing helps explain why item-specific learning in Experiment 2 modulated task conflict, whereas the ISPC manipulation in Experiment 1 did not.

## Limitations and future directions

An alternative explanation for the ISPC effect observed in Experiment 1—namely, the reduction in informational conflict for mostly incongruent items, is that this reduction reflects item frequency or contingency-based learning rather than control adaptation. Extensive prior research has used the confound-minimized ISPC manipulation employed in Experiment 1 and demonstrated through a variety of approaches, including transfer, that frequency-based or contingency-based (and other) accounts cannot explain the ISPC effect that emerges from this design (e.g., Bugg & Hutchison, 2013; Ileri-Tayar et al., 2022, 2024; Suh & Bugg, 2021). A key challenge to these accounts is that the typical ISPC pattern (also observed in Experiment 1) shows an effect of the ISPC manipulation on incongruent but not congruent trials. Accounts based on frequency and/or contingency also predict an effect on congruent trials. The selective speeding on incongruent trials for mostly incongruent items indicates that the manipulation influenced control processes engaged to resolve informational conflict (see Ileri-Tayar, Bugg, et al., 2025a; Suh & Bugg, 2021, for further elaboration).

A similar question could be asked regarding the ISPW manipulation in Experiment 2, particularly with respect to the marker of task conflict, because there were more word trials in the mostly neutral words condition and more nonword trials in the mostly neutral nonwords condition. While we cannot rule out that frequency differences contributed to the modulation of task conflict in this design, two patterns argue against an account based on frequency. First, neutral words were slower in the mostly neutral words condition compared with the mostly neutral nonwords condition^5^, even though they were more frequent in the former condition. Second, and perhaps more tellingly, the ISPW manipulation transferred to frequency-matched markers of informational conflict (e.g., congruency effect). This finding supports the interpretation that the ISPW manipulation led to modulations of task conflict (word reading) that had downstream effects on the response and/or semantic activation of word and color dimensions for congruent and incongruent stimuli. Nonetheless, future studies should include diagnostic items in the ISPW paradigm (e.g., a set of frequency and PW matched neutral words and neutral nonwords) to further isolate the effects of task control.

Another limitation of the present study is that Experiment 1 employed a vocal response modality, whereas Experiment 2 employed a manual response modality. One could argue that the difference in modality explains why task conflict was modulated in Experiment 2 but not Experiment 1. However, this interpretation is unlikely. Prior work suggests that effects of task conflict are more reliably observed with vocal responding than with manual responding (Augustinova et al., 2019; Keha & Kalanthroff, 2023; Kinoshita et al., 2018). Therefore, it is more plausible that the lack of a task conflict effect in Experiment 1 reflects the specificity of reactive adjustments to informational conflict rather than an artifact of modality. In addition to the difference in response modality, a further limitation is that the two experiments differed in length, with Experiment 2 comprising approximately half as many trials overall as Experiment 1, and in principle this could complicate conceptual comparisons between experiments. To evaluate whether the differing findings across experiments may be attributable to differences in trial counts, we reanalyzed Experiment 1 using the same number of trials as in Experiment 2 and obtained the same pattern of results (reported in the Supplementary Materials), suggesting that the key conclusions are not driven by the larger number of trials in Experiment 1. It is also worth noting that our conclusions are grounded primarily in the within-experiment stimulus type by item type interactions rather than in direct comparisons of absolute effect sizes across experiments. In this sense, the experiments are best viewed as addressing two related but distinct questions about item-specific control under different item-level regularities.

A second limitation concerns the level at which informational conflict is resolved. Although we showed that item-specific manipulations of informational control can modulate interference, it remains unclear whether this modulation stems primarily from response competition between alternative responses linked to the stimulus or semantic competition between stimulus dimensions (color vs. word meaning). Future studies could directly address this question by disentangling response conflict from semantic conflict at the item level, in a manner similar to previous approaches that have separately manipulated these sources of conflict (Augustinova et al., 2015; Shichel & Tzelgov, 2018). Doing so would clarify whether reactive adjustments operate at the level of response conflict, stimulus meaning, or both, thereby refining our understanding of the mechanisms through which item-specific control influences performance.

Another potential limitation concerns our operationalization of the nonword baseline. We used nonalphabetic symbol strings to maximize the contrast between readable and nonreadable stimuli and, in turn, to maximize the likelihood that the reading task is engaged when a stimulus is word-like. However, neutral baselines are not interchangeable: prior work shows that different “nonword” controls (symbols, consonant strings, pseudowords, rows of *X*s) can produce meaningfully different patterns, consistent with the idea that “reading” in Stroop tasks reflects multiple component processes rather than a single invariant mechanism (Keha & Kalanthroff, 2023; Kinoshita et al., 2017). Accordingly, our design does not isolate whether the observed task-conflict effects, and their modulation by item-level regularities, are driven primarily by orthographic processing, phonological activation, semantic activation, or their combination. Future studies might systematically vary the type of neutral baseline within the same ISPW manipulation, and test whether reactive control effects are strongest for baselines that differentially engage particular components, thereby clarifying which aspects of the reading task are most critical for eliciting and regulating task conflict in ISPW paradigms.

Finally, although we have described item-specific reactive adjustments in terms of suppression of the word dimension (see Jacoby et al., 2003, for evidence from an ISPC paradigm), enhanced selectivity in the Stroop task can be achieved via reduced distractor (word) processing, amplification of task-relevant (color) processing, or a combination of both. For example, Egner and Hirsch (2005) emphasized target-feature amplification as a mechanism supporting conflict resolution, whereas recent model-based work examining list-wide proactive adjustments suggests that changes are explained primarily by stronger distractor suppression, with weaker evidence for target amplification (Kelber et al., 2025). Together with evidence that item-specific adjustments can be triggered by both task-relevant and task-irrelevant stimulus information (Spinelli et al., 2022), this motivates a more agnostic interpretation of the item-specific effects observed here: they may reflect reduced engagement of word-related processing, enhanced color processing, or both.

## Conclusion

To summarize, Experiment 1 replicated the well-established ISPC effect on informational conflict but yielded no evidence that item-specific control extends to task conflict, suggesting that activation of the word reading task itself was not modulated in this context. In contrast, Experiment 2 showed that when the manipulation directly targeted the probability of encountering readable words (via the ISPW manipulation), item-specific adjustments influenced task conflict, consistent with control over word reading. These adjustments also influenced informational conflict, resulting in modulations of the congruency effect akin to those observed with the ISPC manipulation, although on frequency and proportion congruency matched stimuli. Taken together, our findings indicate that item-specific mechanisms can also regulate the irrelevant reading task, but only when driven by learned probabilities of engaging the reading task rather than by the frequency of conflict between color and word dimensions. In this way, our results refine and extend Jacoby and colleagues’ (2003) idea that the ISPC effect demonstrates automatic control of the word reading task in the color–word Stroop paradigm.

## Supplementary Information

Below is the link to the electronic supplementary material.

Supplementary file1 (DOCX 36 KB)

## Data Availability

All data, materials, and analysis scripts for both experiments are available on the Open Science Framework at: https://osf.io/ybewq/

## Footnotes

1 Another marker of task conflict is the reverse facilitation effect—the first marker of task conflict that appeared in the literature. We preregistered this effect as the primary marker for task conflict. However, in hindsight, following the publication of recent works in the field (Keha & Kalanthroff, 2025a, b), we thought it was more appropriate to treat it as a secondary marker (see Supplementary Materials for analyses of this marker) and the word interference effect as the primary marker. The word interference effect provides a purer index of the conflict between the task of color naming and the automatically triggered task of word reading. This is because unlike the reverse facilitation effect, it does not include two processes operating in opposite directions (i.e., on the one hand, slowing in the congruent condition due to the task of word reading, and on the other hand, speeding in the congruent condition due to the match between the color and word dimensions). In addition, reverse facilitation is typically revealed under situations of reduced proactive control (e.g., where most trials in the block are non-word trials; Entel et al., 2015; Keha, Klotsvog et al., 2024a, Keha, Naftalovich et al., 2024b), when reading is more difficult to inhibit (Goldfarb & Henik, 2007; Kalanthroff et al., 2013). In the current study, we did not try to reduce proactive control, raising the concern that reverse facilitation may not be observed in any condition.

2 For further analysis of additional contrasts, see the Supplementary Materials.

3 Because Experiment 2 was completed online on participants’ own computers, we assessed whether between-participant differences in hardware and software latency influenced the RT findings by rerunning all RT analyses after mean-centering RTs within participants. The pattern of results was unchanged. Full details are reported in the Supplementary Materials.

4 For further analysis of additional contrasts, see the Supplementary Materials.

5 Mean difference = 30, *SE* = 7. This difference was statistically significant, *t*(64) = 4.109, *p* < .001.

## Code availability

All analysis code used in this project is openly available on the Open Science Framework at: https://osf.io/ybewq/
