# Supplementary material for: Item-specific control of word reading as evidenced by a novel proportion wordness effect
Source: Mem Cognit. 2026 Apr 28;54(7):2602–14. doi: 10.3758/s13421-026-01876-4 (PMC13751717; doi:10.3758/s13421-026-01876-4)
Supplement: Supplementary file 1 [file 13421_2026_1876_MOESM1_ESM.docx]

**Supplementary Material**

**Experiment 1 – Error Rate Analysis**

We carried out a 4 × 2 ANOVA for error rate, with stimulus type (congruent, incongruent, neutral words, neutral non-words) and item type (mostly congruent, mostly incongruent) as within-subject factors (see Table S1). Trials that were faster than 200ms were removed from the analysis. This exclusion resulted in removing 617 trials, which is approximately 1% of the total number of trials.

We observed a main effect of stimulus type, *F*(3,171) = 53.121, *p* < .001, $\eta_{p}^{2}$= .482, and conducted post-hoc pairwise contrasts using Benjamini-Hochberg correction. For the information conflict marker, the congruency effect, the error rate was significantly lower for congruent trials compared to incongruent trials, *t*(57) = 9.598, *p* < .001, Cohens’ d = 1.271. For the task conflict marker, the word interference effect, we did not observe a difference between neutral words and neutral non-words, *t*(57) = 1.280, *p* = .999. In addition, no main effect was found for item type, *F*(1,57) = 0.001, *p* = .993.

Finally, and most importantly, we observed a two-way interaction between stimulus type and item type, *F*(3,171) = 2.72, *p* = .046, $\eta_{p}^{2}$= .046. To account for this interaction, we analyzed differences between the two item type conditions (mostly congruent vs. mostly incongruent) in information conflict and task conflict, using a Benjamini-Hochberg correction. The congruency effect did not differ between the mostly congruent (M = 9.1, SE = 1.1) and the mostly incongruent items (M = 6.2, SE = 0.8), *t*(57) = 2.564, *p* = .078. The word interference effect also did not differ between the mostly congruent (M = 0.7, SE = 0.7) and mostly incongruent (M = 0.6, SE = 0.7) items, *t*(57) = 0.051, *p* = .960.

**Experiment 2 – Error Rate Analysis**

We carried out a 4 × 2 ANOVA for error rate with stimulus type (congruent, incongruent, neutral words, neutral non-words) and item type (mostly neutral non-words, mostly neutral words) as within-subject factors (see Table S1). Trials that were faster than 200ms were removed from the analysis. This exclusion resulted in removing 4 trials, which is approximately 0.01% of the total number of trials.

We observed a main effect of stimulus type, *F*(3,192) = 24.503, *p* < .001, $\eta_{p}^{2}$ = .277, and conducted post-hoc pairwise contrasts using a Benjamini-Hochberg correction. The error rate was significantly higher for incongruent trials compared to congruent trials, indicating a congruency effect, *t*(64) = 3.867, *p* < .001, Cohens’ d = 0.483. For the task conflict marker, the word interference effect, there was no significant difference in error rate between neutral non-words and neutral words, *t*(64) = 1.269, *p* = .209.

Most importantly, an interaction was found between item type and stimulus type, *F*(3, 192) = 5.182, *p* = .002, $\eta_{p}^{2}$ = .075. To account for this interaction, we analyzed differences between the two item type conditions (mostly neutral non-words vs. mostly neutral words) with a Benjamini-Hochberg correction. The word interference effect did not differ between the mostly neutral words condition (M = -1.3, SE = 1.9) and the mostly neutral non-words condition (M = 0.4, SE = 0.8), *t*(64) = 1.733, *p* = .132. The congruency effect was significantly smaller in the mostly neutral words (M = 1.4, SE = 1) than the mostly neutral non-words (M = 4.7, SE = 1) condition, *t*(64) = 2.678, *p* = .028).

**Table S1**

***Mean Error Rate and Standard Error as a Function of Stimulus type and Item Type in Experiment 1 and Experiment 2.***

|  | Experiment 1 (N = 58) | | | Experiment 2 (N = 65) | | |
| --- | --- | --- | --- | --- | --- | --- |
|  | Mostly Congruent | Mostly Incongruent | | Mostly Neutral Words | Mostly Neutral Non-words | |
| Congruent | 4.0 (0.4) | 5.5 (0.7) | 8.9 (1.1) | | 4.7 (0.7) |  |
| Incongruent | 13.1 (1.2) | 11.8 (0.7) | 10.4 (1.3) | | 9.4 (1.1) |  |
| Neutral Words | 6.1 (0.6) | 6.0 (0.7) | 6.7 (0.8) | | 3.4 (0.5) |  |
| Neutral Non-words | 5.4 (0.7) | 5.3 (0.6) | 8.0 (1.1) | | 3 (0.4) |  |

*Note*. Means (SE) for error rate for each stimulus type condition in the mostly congruent and mostly incongruent items in Experiment 1, and mostly neutral words and mostly non-words in Experiment 2.

**Additional Analysis
*Decomposition of the Congruency Effect***

In addition, as an exploratory analysis, the current design allowed us to test whether the source of the ISPC effect is driven by interference or facilitation (see Jacoby et al., 2003). In Experiment 1, the interference effect (incongruent – neutral words) was larger for mostly congruent items as compared to mostly incongruent items, t(57) = 5.868, p < .001, Cohen’s d = 0.777. In contrast, the facilitation effect (neutral words – congruent) did not differ between mostly congruent and mostly incongruent items, t(57) = 2.029, p = .057, Cohen’s d = 0.269. In Experiment 2, neither the interference effect, *t*(64) = 1.412, *p =* .163, nor the facilitation effect, *t*(64) = 1.521, *p =* .159, differed between the mostly neutral non-words and mostly neutral words conditions. For further information and decomposition of these effects for error rate see Table S2.

***The Reverse Facilitation Effect — An Additional Task Conflict Marker***

As described in Footnote 1 in the manuscript, we included the reverse facilitation effect as a secondary marker for task conflict. In Experiment 1, we observed a regular facilitation pattern in reaction times, such that the congruent condition was faster as compared to the neutral non-words condition, *t*(57) = 4.708, *p* < .001, Cohen’s *d* = 0.623, and this effect did not differ between the mostly congruent (M = 26, SE = 5) and mostly incongruent items (M = 13, SE = 5), *t*(57) = 2.340, *p* *=* .342, Cohen’s *d* = 0.310. In Experiment 2, we observed a significant reverse facilitation effect such that the congruent condition was slower than the neutral non-words condition, t(64) = 4.740, *p* < .001, Cohen’s *d* = 0.592. In addition, the reverse facilitation effect was smaller in the mostly neutral words items (M = 9, SE = 8) compared to the mostly neutral non-words (M = 50, SE = 8) items, *t*(64) = 3.957, *p* *<* .001, Cohen’s *d =* 0.495. For further information and decomposition of these effects for error rate see Table S2.

***Mean-centered Analysis for Experiment 2***
Since Experiment 2 was conducted online, we repeated the Experiment 2 RT analysis after mean-centering RTs within each participant. Specifically, for each participant, we computed a centered RT score by subtracting that subject’s mean RT across all trials from their RT on each trial. We then conducted the same 4 × 2 repeated-measures ANOVA on centered RT with stimulus type (congruent, incongruent, neutral words, neutral non-words) and item type (mostly neutral words vs. mostly neutral non-words) as within-subject factors. The mean-centered analysis reproduced the original inferential pattern. There was a main effect of stimulus type, F(3, 192) = 109.410, p < .001, $\eta_{p}^{2}$ = .631, and a main effect of item type, F(1, 64) = 48.321, p < .001, $\eta_{p}^{2}$ = .430. Critically, these main effects were qualified by a significant stimulus type × item type interaction, F(3, 192) = 17.418, p < .001, $\eta_{p}^{2}$ = .214. To decompose the interaction, we compared the size of the two conflict markers between item types using the interaction pairwise contrasts with Benjamini-Hochberg correction. The task-conflict marker (word interference effect: neutral words vs. neutral non-words) differed reliably between item types, estimate = 54.8 ms, SE = 8.92, *t*(64) = 6.143, *p* < .001, indicating a smaller word interference effect for mostly neutral-word items than for mostly neutral non-word items. The informational-conflict marker (congruency: incongruent vs. congruent) also differed between item types, estimate = -28.6 ms, SE = 11.30, *t*(64) = -2.544, *p* = .020, indicating a smaller congruency effect for mostly neutral-word items than for mostly neutral non-word items.

***Block analysis in Experiment 1***

Because Experiment 1 was substantially longer than Experiment 2, we conducted an additional block-wise analysis to ensure that the key effects were not driven by time-on-task factors (e.g., practice, fatigue, or gradual strategy shifts) and to provide results that are more directly comparable to the shorter time course of Experiment 2. Specifically, Experiment 1 was divided into 2 blocks (each comprising 432 trials per participant), and block was added as a variable to the RT ANOVA analysis of Experiment 1. Notably, the first half of Experiment 1 (Block 1) was very similar in length to Experiment 2, allowing a more direct comparison of effects early in the task. We then examined whether the characteristic pattern observed in the main analysis differed across blocks.

The stimulus type × item type × block interaction was not significant, *F*(3, 168) = 2.29, *p* = .080, $\eta_{p}^{2}$ = .039. indicating that the ISPC pattern did not reliably change over time. Consistent with this, across both blocks the same robust pattern emerged: the congruency effect was reliably larger in the mostly congruent list than in the mostly incongruent list, indicating a consistent ISPC modulation of informational conflict over time. In contrast, the word-interference effect did not vary as a function of list type in either block, suggesting that the block-wise modulation was specific to the congruency effect and did not extend to word interference. In Block 1, the congruency effect was significantly larger in the mostly congruent list than in the mostly incongruent list, *t*(56) = 3.93, *p* = .001, whereas the word-interference effect (neutral words vs. neutral non-words) did not differ between list types, *t*(56) = 0.82, *p* = .417. The same results were observed in Block 2. The congruency effect was larger in the mostly congruent list than in the mostly incongruent list, *t*(56) = 3.80, *p* = .002, whereas the word-interference effect remained unchanged, *t*(56) = 1.42, *p* = .192.

**Table S2**

***Summary of Mean Differences and t-tests for Pairwise Contrasts (Reaction Time and Error Rate) in Experiment 1 and Experiment 2.***

|  | **Experiment 1**  **(Mostly Congruent -**  **Mostly Incongruent)** | | | | | **Experiment 2**  **(Mostly Neutral Words -**  **Mostly Neutral Non-words)** | | | | |  |  |
| --- | --- | --- | --- | --- | --- | --- | --- | --- | --- | --- | --- | --- |
| **Contrast** | Reaction Time | | Error Rate | | Reaction Time | | | Error Rate | |  |  |  |
| Interference Effect (Incongruent – Neutral Words) | 39.39 (6.71) [t = 5.868, p < .001] | -1.28 (1.17) [t = 1.089, p = .373] | | | -14.4 (10.20) [t = 1.412, p =.163 ] | | | 3.25 (1.21) [t = 2.678, p = .028] | | | |  |
| Facilitation Effect (Congruent – Neutral Words) | -13.50 (6.65) [t = 2.029, p = .057] | 1.63 (0.76) [t = 2.144, p = .083] | | | 14.3 (9.37) [t = 1.521, p = .160] | | | -1.65 (0.95) [t = 1.733, p = .132] | | | |  |
| Reverse Facilitation Effect  (Congruent – Neutral Non-words) | -13.12 (5.61) [t = 2.340, p = .034] | | | 1.59 (0.76) [t = 2.085, p = .083] | | | 40.5 (10.20) [t = 3.957, p < .001] | | -0.78 (0.94) [t = 0.823, p = .413] | | | |

*Note*. The table presents pairwise comparisons conducted to further examine the interaction patterns in each experiment. These contrasts are additional contrasts to the two markers of information conflict and task conflict that were reported in the manuscript. For Experiment 1 (mostly congruent vs. mostly incongruent items), the contrasts quantify differences in both reaction times (in milliseconds) and error rate (%) between incongruent, congruent, and neutral stimulus types (neutral words and neutral non-words). For Experiment 2 (mostly neutral words vs. mostly neutral non-words), analogous contrasts were computed to assess performance differences across the same stimulus type conditions. Values represent mean differences along with their standard errors (in parentheses), followed by the corresponding t- and p-values obtained from pairwise post-hoc tests. All p-values were adjusted using the Benjamini–Hochberg procedure to control for multiple comparisons.

***Table S3***

***Mean Reaction Times and Standard Error as a Function of Stimulus type and Item Type in Experiment 1 and Experiment 2.***

|  | Experiment 1 (N = 58) | | Experiment 2 (N = 65) | | |
| --- | --- | --- | --- | --- | --- |
|  | Mostly Congruent | Mostly Incongruent | Mostly Neutral Words | Mostly Neutral Non-words | |
| Congruent | 579 (9) | 581 (10) | 712 (15) | 667 (15) |  |
| Incongruent | 730 (14) | 679 (11) | 797 (18) | 781 (18) |  |
| Neutral Words | 640 (11) | 628 (12) | 699 (14) | 669 (14) |  |
| Neutral Non-words | 605 (10) | 594 (10) | 702 (15) | 617 (12) |  |

*Note.* Means (SE) for reaction times (RT) for each stimulus type
